# What drives seed dispersal effectiveness?

**DOI:** 10.1002/ece3.10459

**Published:** 2023-08-31

**Authors:** Omer Nevo, Caitlynn Filla, Kim Valenta, Eugene W. Schupp

**Affiliations:** ^1^ German Centre for Integrative Biodiversity Research (iDiv) Halle‐Jena‐Leipzig Leipzig Germany; ^2^ Institute of Biodiversity Friedrich Schiller University Jena Jena Germany; ^3^ Department of Anthropology University of Florida Gainesville Florida USA; ^4^ Department of Wildland Resources and Ecology Center Utah State University Logan Utah USA; ^5^ Estación Biológica de Doñana (EBD‐CSIC) Integrative Ecology Group Sevilla Spain

**Keywords:** animal–plant interactions, forest regeneration, frugivory, seed dispersal

## Abstract

Seed dispersal is a critical phase in plant reproduction and forest regeneration. In many systems, the vast majority of woody species rely on seed dispersal by fruit‐eating animals. Animals differ in their size, movement patterns, seed handling, gut physiology, and many other factors that affect the number of seeds they disperse, the quality of treatment each individual seed receives, and consequently their relative contribution to plant fitness. The seed dispersal effectiveness framework (SDE) was developed to allow systematic and standardized quantification of these processes, offering a potential for understanding the large‐scale dynamics of animal–plant interactions and the ecological and evolutionary consequences of animal behavior for plant reproductive success. Yet, despite its wide acceptance, the SDE framework has primarily been employed descriptively, almost always in the context of local systems. As such, the drivers of variation in SDE across systems and the relationship between its components remain unknown. We systematically searched studies that quantified endozoochorous SDE for multiple animal species dispersing one or more plant species in a given system and offered an integrative examination of the factors driving variation in SDE. Specifically, we addressed three main questions: (a) Is there a tradeoff between high dispersal quality and quantity? (b) Does animal body mass affect SDE or its main components? and (c) What drives more variation in SDE, seed dispersal quality, or quantity? We found that: (a) the relationship between quality and quantity is mediated by body size; (b) this is the result of differential relationships between body mass and the two components, while total SDE is unaffected by body mass; (c)neither quality nor quantity explain more variance in SDE globally. Our results also highlight the need for more standardized data to assess large‐scale patterns in SDE.

## Graphical abstract

Seed dispersal effectiveness (SDE) is a quantitative framework designed to quantify the contribution of animals to plant fitness. It is widely accepted but is mostly applied locally. We analyze SDE data from dozens of publications to assess what factors drive variation in SDE across systems.

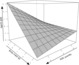

## 1 INTRODUCTION

Seed dispersal is a major part of plant reproduction and drives succession patterns, regeneration, and plant spatial‐genetic structure (Gelmi‐Candusso et al., 2017; Howe & Miriti, 2004). A substantial proportion of angiosperms have evolved to pack their seeds in a nutritious tissue that attracts fruit‐eating animals to ingest the seeds and disperse them away from the parent. This dispersal strategy (endozoochory) is present in a third to almost half of woody species in temperate regions and in about 90% of woody plants in tropical rainforests (Herrera, 2002). The latter pattern is thought to result from the strong competition for light in dense tropical forests, which selects for larger and more energy‐rich seeds that are in turn more effectively dispersed by animals compared to abiotic dispersal strategies (Bolmgren & Eriksson, 2005, 2010). The expanding availability of fleshy fruits has created a broad niche space for fruit‐eating animals, resulting in a tremendous diversification of fruit‐eating animals from many different clades, for example, birds, primates, bats, rodents, and many others (Eriksson, 2016).

Rarely, plant–frugivore interactions are highly specialized (Chapman et al., 1992; Reid, 1991). However, in most communities, frugivores and fruit‐bearing plants form generalist interaction networks in which frugivores consume the fruits of a few to many plant species and plant species receive dispersal services from multiple frugivores (Blüthgen et al., 2007). Yet while broadly conceived as mutualists, seed dispersing animals are not altruists—they are selected to maximize their own fitness rather than plant fitness (van Leeuwen et al., 2022). The net effects of frugivores on the reproduction of animal‐dispersed plants are situated along a continuum, as frugivore species differ in effects, for example, in the number of seeds dispersed and the seed treatment they provide (Marques Dracxler & Kissling, 2021; Schupp et al., 2010). The relative effect of a frugivore species on plant fitness results from a complex combination of physiological, behavioral, and demographic factors that vary among dispersers. The variation in frugivores' contribution to plant recruitment is critical for understanding selection pressures on plant traits (Gómez et al., 2022; Valenta et al., 2018), as well as presenting interactions that can inform conservation efforts (McConkey et al., 2012). As such, the differential fitness benefits provided by different animals are relevant for understanding the past in terms of evolutionary history, the present in terms of regeneration patterns, and the future in terms of potential responses to global change.

Despite its importance, the net effect of frugivores on plant fitness is almost impossible to measure, as it would, in principle, require monitoring seed fate from removal to development into a reproductive adult (Schupp, 1993). As such, a common approach has been to rely on various incomplete and often non‐comparable proxies. In 1993, Schupp (1993) introduced a quantitative framework for evaluating seed dispersal effectiveness (SDE), which was later refined (Schupp et al., 2010). This framework allows for the systematic combination of the multitude of factors determining the net effect of a seed disperser species or guild on the fitness of a plant species (Schupp, 1993; Schupp et al., 2010). It explicitly defines two major components: seed dispersal quality and quantity. Quality is defined as the probability that each individual dispersed seed will survive, germinate, and establish, and quantity as the number of individual seeds dispersed by, for example, a single frugivorous species. Each component is determined by multiple factors; for example, quantity is a function of population density, body mass, and the degree of frugivory. Quality is a function of factors like gut treatment, deposition site, etc. A multiplication of quantity and quality leads to total SDE, which serves as a proxy for the overall contribution of animals to plant reproductive success (Schupp, 1993; Schupp et al., 2010).

Since its inception, the SDE framework has been a major organizational and empirical tool that has been instrumental in the standardization of terminology and methods in seed dispersal research (Jordano et al., 2007). Numerous local studies have relied on the components of SDE to evaluate the degree to which different species or guilds contribute to local plant fitness (Fuzessy et al., 2016; Godínez‐Álvarez, 2004; Traveset & Verdú, 2002; Valenta & Fedigan, 2008). Yet with few exceptions (Godínez‐Alvarez et al., 2020), most studies started and remained descriptive at the local community level, thus leaving the original goals of high‐level synthetic understanding of the effects of seed dispersers on plant fitness unaddressed (Gómez et al., 2022).

This particularly manifests in several knowledge gaps with regards to the “big picture” of SDE. The first is the nature of the relationship between the two components of SDE—quality and quantity. While theoretically and mathematically independent in the original SDE framework, ecological reality dictates that the two may, in some cases, be affected by similar mechanisms. For example, high quantity that is driven by the large body mass of an animal might be predicted to be associated with low quality because seeds are released in large clumps and thus potentially face high density‐dependent mortality (Connell, 1971; Janzen, 1970). However, if high quantity is a function of a high abundance of highly frugivorous small animals, the relationship between quality and quantity is not expected to be negatively correlated. In extreme cases, high dispersal quality by a particular group of frugivores could even generate selection to exclude others, thus generating a positive correlation between the two components. In this context, a recent analysis suggested that in birds, body mass drives variation in SDE, following an inverted U‐shaped relationship in which certain body sizes provide an optimal SDE (Godínez‐Alvarez et al., 2020). Yet this was based on the mathematical constructs of SDE rather than fully empirical data.

Another open question regarding the relationship between quality and quantity is whether, and in what contexts, either of them tends to explain more of the variance in total SDE—is variation in SDE driven more by the quantity component, the quality component, or by both components equally (Gómez et al., 2022)? For example, if the quantity component is very low, changes in the quality component are expected to have little impact on SDE—SDE is very low, driven substantially by the quantity component (Gómez et al., 2022; Schupp et al., 2010). There could be systems in which most animals provide comparable quality and variance in SDE is driven primarily by the amount of seeds each animal disperses, or the exact opposite. If any pattern exists, it may be global or it may be the result of a particular network structure. In this context, a recent analysis suggested that a positive component correlation is expected to be present in systems where quality explains most variance in SDE and vice versa (Gómez et al., 2022), but this was found in an analysis of synzoochorous systems (where seeds are not wrapped in a nutrition tissue and are not ingested) rather than the more common endozoochorous systems, which rely on nutritious fleshy fruits.

Here, we synthesize studies quantifying SDE from around the globe to identify broad patterns in SDE across systems. We identified 38 studies that explicitly measured SDE or its components for multiple animal species on single or multiple plant species (or pooled groups) and assessed three sets of questions. Given that SDE is standardized and well defined *within* individual studies but not among studies (e.g., different proxies are used to assess the quality component), we standardized all values within a given study by z‐transforming them (mean = 0, SD = 1). We then tested our questions primarily using a mixed‐effects model approach. This analytical approach did not seek to directly compare studies, but rather to identify whether the different studies consistently showed similar trends despite using somewhat different methods. Specifically, independent of differences among studies in how quantity, quality, and SDE were measured, do we tend to find the same within‐study trends repeatedly occurring across the different studies with respect to the following questions:

Q1. Are the quantity and quality components of SDE correlated, and is this correlation driven by variation in body mass?
Q2. Is body size a main driver of SDE or its components?
Q3. What explains more variance in SDE, the quality or quantity component? Is positive component correlation associated with higher importance of the quality component and vice versa (Gómez et al., 2022)?

## 2 MATERIALS AND METHODS

### 2.1 Search criteria and data collection

A systematic search of all studies quantifying SDE was conducted using the Web of Science database. The initial search targeted all papers that have cited the Schupp (1993) and/or Schupp et al. (2010) SDE framework publications. This search was conducted in June 2021. Two other search parameters were then employed to identify potentially relevant studies that may not have cited the original framework papers: (1) a keyword search for “seed* dispers* effectiveness” OR “seed* dispers* efficiency”; and (2) a search for all papers citing any paper found in the original search that had themselves been cited more than 200 times. Finally, the resulting list of publications was compared to the list of publications included in Godínez‐Alvarez et al. (2020). The original search for papers citing Schupp (1993) and/or Schupp et al. (2010) identified almost all relevant studies. Altogether, our initial publication list included 1190 manuscripts (see full workflow in Data S1).

**FIGURE 1:**
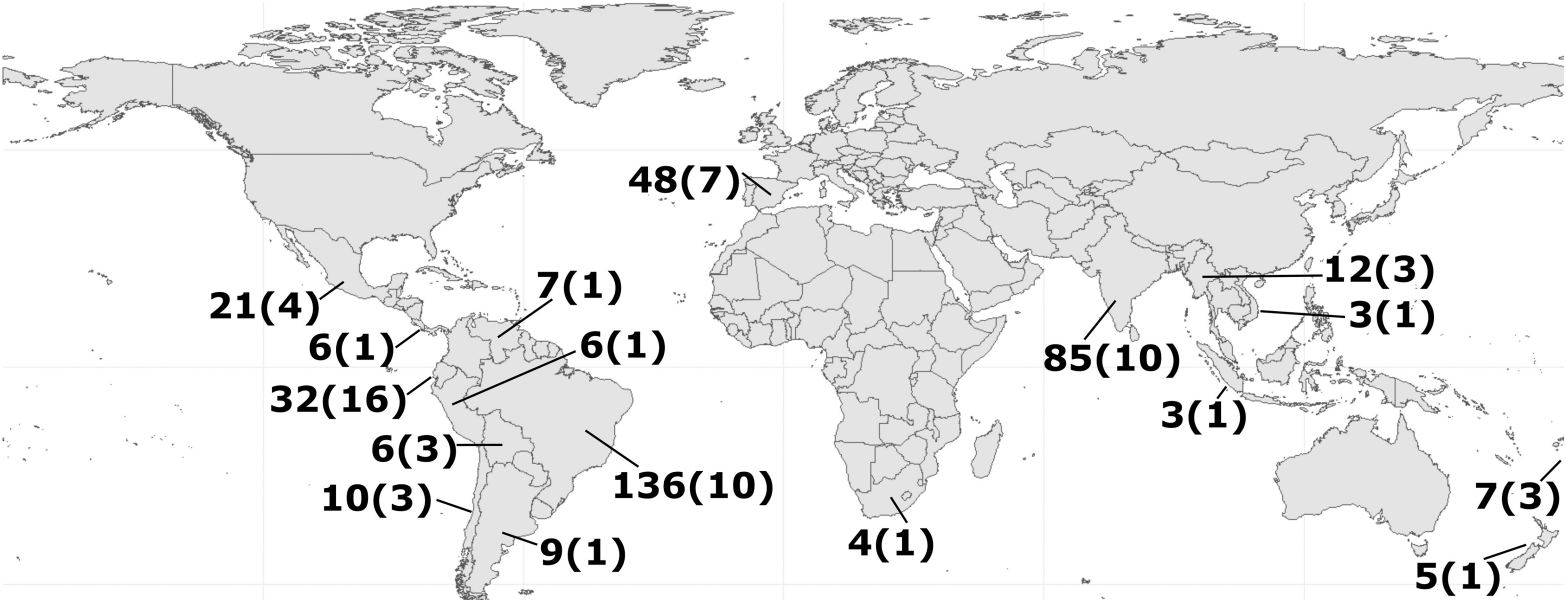
Distribution of study species and data points used in the final analyses. Numbers outside the brackets represent the number of data points, that is, a single SDE or component value for an animal dispersing a plant or a pooled group of plant species. Numbers in brackets represent the number of plant species (or pooled species) for which SDE or its components were quantified. The figure notes only studies that were used at least once in one of the analyses and excludes studies that were filtered out (see Section 2).

We systematically read all studies included in the initial list to identify those that explicitly quantified SDE or close proxies and consecutively included only studies that quantified SDE or its components in multiple animals on a single plant (or pooled plant species). From these papers, we extracted the total SDE, its components (seed dispersal quality, seed dispersal quantity), disperser and plant species, disperser body mass, disperser guild, habitat type, and any quantity, or quality subcomponents. SDE was, in most cases, a simple multiplication of the quantity and quality components. In some studies, raw figures were further altered (e.g., multiplying by 100 to convert a fraction to a percentage), but this had no effect on the mathematical relationship between the numbers and was particularly irrelevant for the analysis here because all values were scaled (see below). For studies that presented SDE, quality, and quantity only as data points in figures, we used Plot Digitizer (http://plotdigitizer.sourceforge.net/) to acquire values. In cases where more than one value was calculated for a species pair (e.g., animal A with plant X), we calculated the mean values for the unique pair and used this as a data point (see R code in Data S1). The full dataset, including all publications identified at each stage and traits not used in the final analysis, is available as an Supporting Information. The final analyses all used subsets of this dataset (described below) and were together based on 400 data points from 67 plant species (or pooled species) from 17 countries (Figure 1).

### 2.2 Data processing

We classified animal dispersers into seven major functional groups: birds, rodents, ants, bats, ungulates, and other non‐volant mammals (primates, foxes, etc.). The latter was based on functional similarity and included all non‐volant mammals, excluding very large ones such as bears or ungulates, as well as non‐frugivorous seed dispersers like squirrels or rats. For brevity, we refer to them as “non‐volant mammals” throughout the manuscript. This was done because functionally, primates are not clearly distinct from other mammals in terms of body mass, habitat type, or other factors, and also to ensure a sample size sufficient for the analyses conducted. Our dataset also included a handful of data points in which dispersers could not be classified into any of the major guilds (e.g., beetles, crabs), which were excluded from the relevant analyses. We obtained body mass for all animals either from the original publications or from EltonTraits 1.0 (Wilman et al., 2014). Data for the same frugivore species feeding on the same plant species in the same study were averaged. All quantitative variables were then z‐transformed (mean = 0, SD = 1) for comparability among studies.

### 2.3 Statistical analysis

SDE, quality, and quantity were quantified differently in each study, making them not directly comparable. As such, our approach was to assess *within study* patterns and then observe to what degree those patterns are consistent across studies.

To test whether SDE quality and quantity are positively or negatively correlated (Q1), we used SDE data from 26 studies and 42 plant species (or pooled species) (total 168 data points). We used Bayesian mixed effects models (bLMMs) from the R package blme (Chung et al., 2013). Most studies calculated SDE for a single plant species, but a handful calculated SDE for a frugivore community on more than one plant species or pooled data for multiple plant species. To account for differences in methodology, all bLMMs included plant species within study (henceforth “study‐plant species”) as an intercepts‐only random effect. The latter allowed us to assess random variance originating from between‐study variation in methodology etc., and focus on variance *within* each study. With no a priori hypothesis, we also originally included random slope models. These, however, proved unstable (they failed to converge in most cases) and hence unreliable. *p*‐Values were calculated using a likelihood ratio test using a Chi‐square distribution with one degree of freedom to compare the full model to a nested null model excluding the relevant predictor. Model assumptions were verified using *qq*‐plots, residual histograms, and scatterplots of residuals versus fitted values. Like many regression models, bLMMs may underestimate slopes with increased uncertainty in the measurement of the predictor variable; for this reason, we do not interpret effect size per se and focus on *p*‐values to estimate whether a significant relationship between two variables is supported by the data. We ran a bLMM modeling quantity on quality in all guilds for which we had at least 12 data points (non‐volant mammals—17; birds—102; rodents—12; ants—25). Because all the data for ants came from a single study, we used a comparable non‐mixed model with identical terms but excluding the random factor. Given the large sample size for birds and the relatively large variation in body size among them, for birds we also ran a bGLM modeling SDE quality on SDE quantity interacting with body size. We calculated the conditional *R* ^2^ based on (Nakagawa & Schielzeth, 2013). To test the relationship between body mass and SDE (Q2), as in previous analyses, we relied on within‐study variation. Sample size permitted only analyses of birds (272 data points). We used a bLMM to model SDE, quality, and quantity against body mass. SDE was analyzed alone. We also ran quadratic models to confirm recently published results (Godínez‐Alvarez et al., 2020). Because body mass appeared to mediate the relationship between quality and quantity, we first ran a model of body mass on quality and quantity with an interaction term between the two. Since the interaction term was not significant, we removed it and then moved on to test whether quality or quantity are correlated with body mass independently of one another by including both as fixed factors in the model. *p*‐values and model assumptions were dealt with as described for Q1.

To estimate whether SDE quality or quantity systematically explains more of the variance in total SDE (Q3), we ran two linear models on each study‐plant species, one in which SDE was modeled only on quality and the other only on quantity. We then extracted the *R* ^2^ from each model and ran a Mann–Whitney test to test if the variance explained by either was systematically larger. We further tested whether the correlation between SDE quality and quantity across studies tends to be associated with either a higher contribution of the quality or quantity components. To this end, we calculated the correlation coefficient between SDE quality and quantity in each study, as well as the ratio of *R* ^2^ (quality: quantity). For this analysis, we included only studies with >3 data points to avoid those in which *R* ^2^ = 1 by definition. This analysis was therefore based on 19 study plant species with a median of five data points per study.

All analyses were conducted on R 4.2.1 (R Core Team, 2014) and the packages blme (Chung et al., 2013), lme4 (Bates et al., 2015), readxl (Wickham & Bryan, 2022), MuMIn (Bartoń, 2022), plot3D (Soetaert, 2014), and dichromat (Lumley, 2022). Data and code are available as Data S1.

## 3 RESULTS

### 3.1 Q1—are quality and quantity components correlated?

**FIGURE 2:**
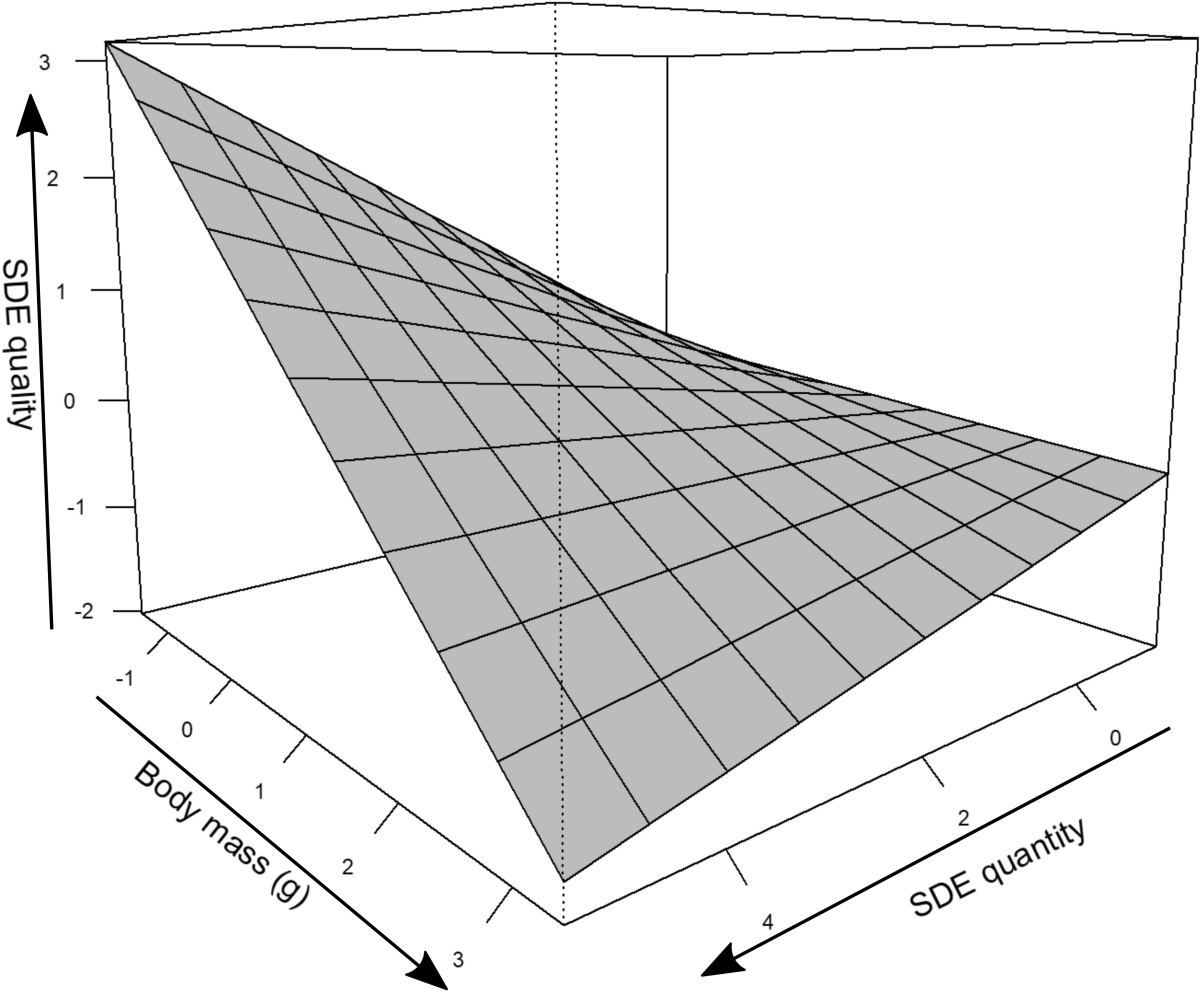
The relationship between seed dispersal effectiveness, quality, and quantity is affected by body mass in birds. The curved surface shows model predictions of the bGLM, averaging the random intercept across studies. The model indicates that among large‐bodied birds, quality and quantity are negatively correlated, while in small‐bodied birds, quality and quantity are positively correlated. A second version of the plot, including data points, is available as Figure S1.

When analyzed independently, we did not find a significant relationship between SDE quality and quantity in any disperser group (non‐volant mammals: *χ* ^2^ = 0.98, df = 1, *p* = .32; birds: *χ* ^2^ = 0.18, df = 1, *p* = .58; ants: *χ* ^2^ = 0.99, df = 1, *p* = .2; rodents: *χ* ^2^ = 0.43, df = 1, *p* = .51). Given the larger body mass range in birds, the adequate sample size with birds, and past findings that body mass may play a role in SDE (Godínez‐Alvarez et al., 2020), we further tested whether body size mediates the relationship between quality and quantity within birds. Modeling SDE quality, there was a significant interaction between body size and seed dispersal quantity in which the quality‐quantity relationship is negative in larger birds but positive in smaller ones (*χ* ^2^ = 4.27, df = 1, *p* = .039; Figure 2). The model explained 15% of the variance, indicating that much variance is explained by other factors.

### 3.2 Q2—is body size driving lower SDE, SDE quality, or SDE quantity

**FIGURE 3:**
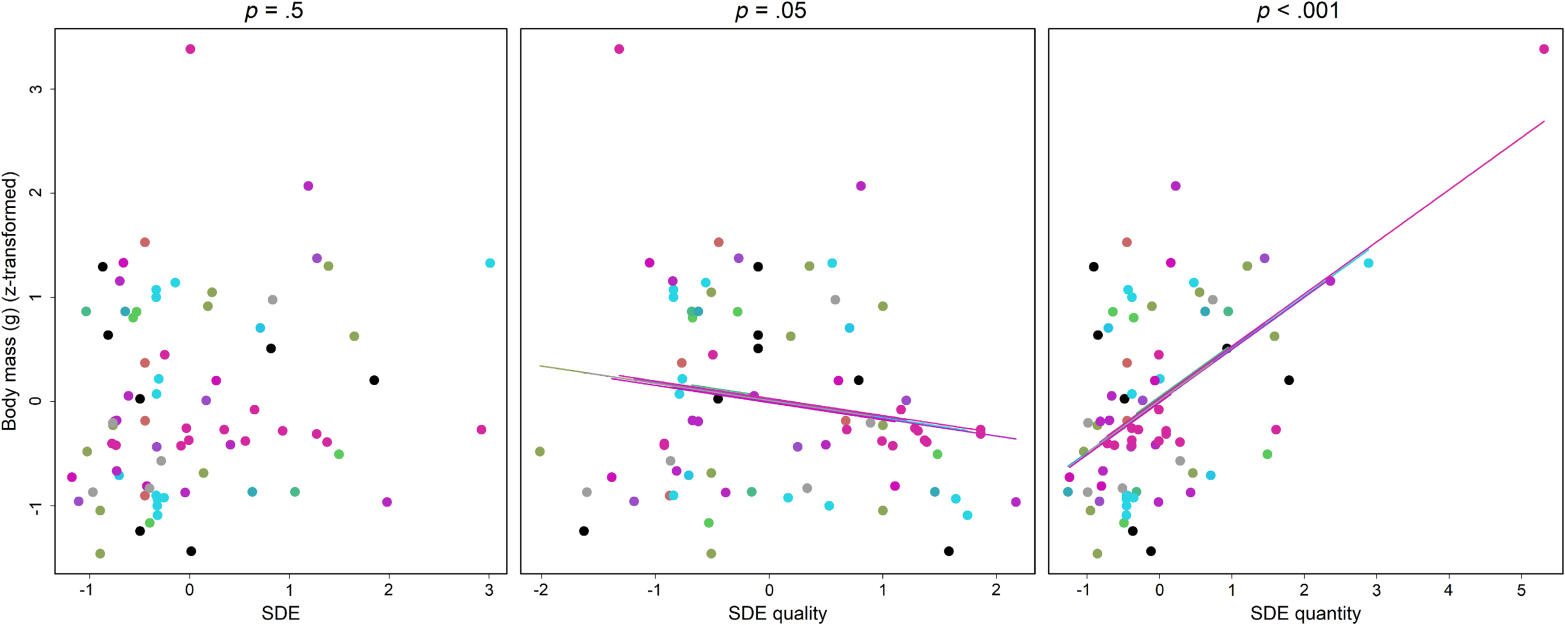
The relationship between body mass and seed dispersal effectiveness (SDE), quality, and quantity in birds. *X* axis—body mass, *z*‐transformed. *Y* axes—SDE, SDE quality, SDE quantity, all *z*‐transformed. *p* Values are from Bayesian mixed effects model (see Section 3). For quality and quantity, they derive from a single model that includes both, thus providing the effect of each independently of the other. Each dot represents a single interaction (a bird species with a plant species or a pool of species). Colors represent plant species (or a pool of species). Note that the almost full convergence of the regression line intercepts, indicating low variance of the random effect, is likely enhanced by the fact that the data for each study was centered by setting the mean to 0 (see Section 2). Note that the choice of axes does not represent typical causality (where x is “independent” and y “dependent”). Axes were selected based on the models where, in order to assess independent relationships, body mass was a response variable. Also note that these are type 1 models (where residuals are parallel to the *y* axis), which may, in cases where the predictors are measured rather than experimentally controlled, inflate the slope but not the direction or its statistical significance. Regression lines should therefore be seen as an indication of the directionality of the relationship between variables, not an exact estimate of the effect size.

Total SDE was not correlated with body mass in birds (*χ* ^2^ = 0.92, df = 1, *p* = .34). We further ran a polynomial model to replicate the results by Godínez‐Alvarez et al. (Godínez‐Alvarez et al., 2020), but again found no relationship between SDE and body mass (*χ* ^2^ = 1.37, df = 1, *p* = .5). Independent of one another, seed dispersal quantity was positively correlated with body mass in birds (*χ* ^2^ = 29.96, df = 1, *p* < .001), while quality was marginally negatively correlated with body mass (*χ* ^2^ = 3.74, df = 1, *p* = .05) (Figure 3).

### 3.3 Q3. What explains more variance in total SDE—quality or quantity?

The mean *R* ^2^ of quality models was 0.5 (SD = 0.36) as opposed to 0.69 (SD = 0.37) for quantity (note that the sum can exceed 1 due to correlation). This difference was not statistically significant (Mann–Whitney test: *W* = 124.5, *p* = .11), indicating that in general, SDE does not tend to be driven more by either of its two main subcomponents.

**FIGURE 4:**
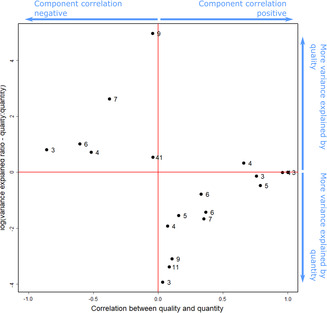
The relationship between the correlation between seed dispersal effectiveness (SDE) quality and quantity (*x* axis) and the relative contribution of each component to total SDE (*y* axis). Each data point is a single study‐species for which at least three data points were available to calculate the correlation between the components and their relative contribution. *X* axis: correlation coefficient between SDE quality and quantity (measured as *R* ^2^ of a linear regression model). *Y* axis—the ratio of proportions of variance explained by quality and quantity (quality/quantity), log transformed. A vertical line separates studies with positive and negative correlation between SDE quality and quantity. The horizontal line separates studies in which quality explained more or less than quantity (since log(1) = 0). Upper left square: negative correlation, higher contribution of SDE quality. Upper right square: positive correlation, higher contribution of SDE quality. Bottom right square: positive correlation, lower contribution of SDE quality. Bottom left square: negative correlation, lower contribution of SDE quality. The numbers to the right of each data point are the sample size for the corresponding plant‐study species.

Examining (a) the association between quality and quantity across studies and (b) the degree of contribution of each subcomponent to total SDE revealed a clear pattern. Studies almost entirely combined either a negative correlation between quality and quantity and a high relative contribution of quality (upper‐left quadrant in Figure 4) or, in direct contrast, a positive correlation between quality and quantity with a high relative contribution of quantity (lower‐right square in Figure 3). At the same time, these distinct clusters showed clear structure: the further away from no correlation between the SDE quality and quantity (extreme left or right in Figure 4), the more balanced the variation explained by the quality and quantity components (i.e., *Y* values closer to zero in Figure 4).

## 4 DISCUSSION

Our objective was to synthesize all studies quantifying SDE across multiple dispersers to address three “big picture” questions around SDE by animals. Given that SDE, quality, and quantity are not fully standardized across studies, we scaled the values within each study and used a mixed‐effects model approach to identify repeating patterns. We tested (Q1) whether the main subcomponents of SDE are independent of one another; (Q2) whether body mass in birds explains SDE, quality, or quantity; and (Q3) whether any of the two components explains more variance in total SDE.

### 4.1 Q1—are the quality and quantity components independent?

The effect of disperser guild on the component correlation was at first ambiguous. The true nature of this effect was revealed only when including body mass in the analysis, which, due to the small available sample size, was only possible for birds. This analysis revealed that among birds, larger‐bodied dispersers had a negative component correlation, while smaller‐bodied species had a positive component correlation. This is in line with the hypothesis that a positive correlation between seed dispersal quality and quantity is a feature of more specialized systems (Gómez et al., 2022), since larger birds appear to be more generalists in the sense that they tend to consume and disperse a larger number of fruit species than do smaller birds (García, 2016; García et al., 2014; González‐Castro et al., 2015; Olesen et al., 2010; Palacio et al., 2016; Wheelwright, 1985). Small sample sizes did not allow testing this hypothesis for other functional groups. However, we expect this relationship to be weaker or absent in other groups since, as opposed to birds, in which size (and hence gape width) are a strong filter and drive specialization (Palacio et al., 2016; Wheelwright, 1985), most other dispersers are less limited by fruit size and can rely on a broader range of fruits. If this is the case, it can be predicted that the tradeoff between quality and quantity is larger in bird‐dominated systems (e.g., temperate regions) but not, for example, in systems where primates provide most dispersal services, like Madagascar.

### 4.2 Q2—is body size negatively associated with total SDE, quality, or quantity

We tested whether body mass is a likely predictor of SDE. Body size is expected to positively scale with fruit/seed intake (the number of seeds dispersed per visit subcomponent, and hence, all else being equal, seed dispersal quantity), but also generally result in larger fecal clumps containing conspecific seeds, thus increasing density‐dependent mortality and reducing seed dispersal quality. Given the small sample size of all other frugivore guilds, this analysis was limited to birds—a group in which this question has been partially addressed in a recent meta‐analysis (Godínez‐Alvarez et al., 2020).

The results of the bird study are generally in line with our expectations: larger birds disperse more seeds, but they may also tend to provide somewhat lower seed dispersal quality; however, overall SDE is not affected by body size. In fact, any other result would be surprising. In birds, there is a unique link between body and fruit/seed size (Galetti et al., 2013; Wheelwright, 1985), and, at least in one system, plants have been shown to rapidly respond to changes in bird size, taking only a few decades to show evolutionary change in response to the local disappearance of large frugivorous birds (Galetti et al., 2013). In this case, if larger birds systematically provide a higher overall SDE, we would expect to see evidence of strong selection for larger fruits, similarly to fruits specializing on large primates (Valenta et al., 2020).

These results are interesting to examine in light of a recent publication that addressed similar questions using a similar approach. Exploring the subcomponents of the main SDE components (i.e., factors driving variation in seed dispersal quality or quantity), Godínez‐Alvarez et al. (2020) found that seed removal is positively associated with bird body size and gut retention time is positively correlated with body mass. These results are in line with our findings. Yet interestingly, our results regarding total SDE differ from theirs: we found no association between SDE and bird body size, whereas they reported a very strong inverted‐U relationship in which the highest SDE is provided by mid‐sized birds. The most likely explanation for the discrepancy is the difference in how SDE was measured in these studies. SDE in Godínez‐Alvarez et al. was not empirically measured. Rather, it was a statistical construct generated by multiplying the *predicted* values from regression models, using only models that found a significant relationship between SDE subcomponents and body mass while not including those that did not. This construction necessitates a tight relationship between SDE and body mass. As such, this approach has strong value in generating hypotheses, namely what the relationship between SDE and body mass should look like given the relationship between the components and body mass. Yet it is nonetheless a hypothesis that requires testing. Our analysis focused on empirically measured SDE across study sites, and our results failed to find this putative relationship between SDE and body size.

### 4.3 Q3. What explains more variance in total SDE—quality or quantity?

Overall, quantity and quality did not differ in the proportion of variance in SDE explained. This implies that *across systems*, the two components tend to contribute similarly. However, this obscures a more interesting and complex pattern, in that whether quantity or quality explains more SDE variance is largely dependent on whether the component correlation is positive or negative. Specifically, nearly all studies clustered into either a group with negative component correlation and a greater contribution of quality to total SDE (upper left quadrant, Figure 4) or a group with positive component correlation and a greater contribution of quantity to total SDE (lower right quadrant, Figure 4), with almost no studies in the other quadrants. Although the analytical approaches are different, this result contrasts with the expectations and results of a recent analysis of a very large database on synzoochorous seed dispersal interactions (Gómez et al., 2022). Those authors suggested that (a) more specialized dispersal systems would have quality‐driven effectiveness and a positive component correlation, while (b) more generalized dispersal systems would have quantity‐driven effectiveness and a negative component correlation, combinations that are nearly absent in the present study. Further, they found that in the synzoochorous dispersal system, as expected, there was significant positive component correlation coupled with quality‐driven effectiveness, suggesting a pathway to specialization driven by the quality component.

Beyond the larger‐scale pattern of nearly all interactions being in either the upper left or bottom right quadrants, the actual distribution of the points within quadrants is intriguing (Figure 4). These patterns strikingly resemble a $y=-\frac{1}{x}$ function in which values further away from the *x*‐axis origin (0) are approaching the *y*‐axis origin (0), and vice versa; or alternatively, a $y=-\frac{1-x}{x}$ function in which the *x*‐axis asymptotes approach 1 on both ends. In either case, these results suggest that the lower the component correlation between quality and quantity is (i.e., the more independent they are from one another), the more the system is driven by quantity or quality. To some degree, this result is not surprising given the construction of the variables: in systems in which SDE quality and quantity are highly correlated (negatively or positively), they will tend to explain a similar amount of the variance and be closer to the *y* = 0 asymptote. In contrast, the much higher variance when the correlation is low (around *x* = 0) and especially the clear directionality (more relative contribution of quality to its left, less to its right) are not necessitated by the math (although they might also be affected by the fact that both derive from the variance in each component and their covariance) and may indicate an ecological phenomenon. Regardless, this pattern indicates that the relationship between quality and quantity is somewhat constrained: an increase in the variance in one, which leads to the other driving most variance in SDE, leads to a growing disassociation of the two. Beyond that, it is left for future studies to explore what drives systems to high dominance of either component in these situations, as well as what factors may drive systems away from this pattern.

### 4.4 Synthesis, caveats, and recommendations for future studies

The three questions we put forward naturally divide into two main categories—Q1 and Q3 deal with the relationship between the main components of SDE, while Q2 relates to variables driving variation in SDE across and within frugivores.

The combined results of Q1 and Q3 indicate that, while complicated, the relationship between SDE quantity and quality may follow predictable patterns. At least in birds, the correlation between the two subcomponents is associated with body mass, and this in turn is associated with a higher or lower contribution of each component to total SDE. In our analysis, the implication is that in dispersal networks involving smaller birds, seed dispersal quality and quantity would tend to be positively correlated (Figure 2). Systems with a positive correlation between quality and quantity tend to also have a higher proportion of SDE explained by the quantity component (Figure 4, bottom‐right quadrant). As such, it is possible that in these systems, on average, the quality of dispersal is similar among small birds, and total SDE is more affected by the quantity of seeds they are able to disperse. In contrast, systems with larger birds where the correlation between seed dispersal quality and quantity is negative (Figure 2) would on average see a higher proportion of SDE explained by the quality component (Figure 4, upper‐left quadrant), indicating that in those systems dispersal quantity is less of a limiting factor than quality. Not surprisingly, these conclusions are also in line with the results from Q2, which identified that in birds, large body size tends to increase seed dispersal quantity and decrease seed dispersal quality.

It is critical to note that despite the fact that the SDE framework is soon to celebrate its 30th anniversary and that it has been highly influential, the number of studies able to address the questions we raised here is still rather small and also geographically limited to mainly tropical and subtropical regions (Figure 1). This is particularly true for Q2, in which data from species other than birds was scant. This inhibits our ability to directly compare the dispersal services of different frugivores within and between guilds. Yet understanding this variation is critical for our understanding of contemporary ecological systems, restoration and conservation efforts, and evolutionary inferences. We therefore believe that this synthesis is far from the final word on the questions we raised here. More data on more species from more diverse systems, particularly underrepresented frugivore guilds, is required, and when they become available, the questions we asked and patterns we identified need to be reevaluated. In addition, our classification into functional groups (e.g., “terrestrial mammal”), while based on anatomical, sensory, and phylogenetic considerations, is not the only reasonable way to split the dataset. Our own results in birds show that within‐group variance plays a major role in affecting SDE and its components. More data in the future will allow replacing the cruder classification with a quantitative classification based on functional traits, thus unearthing the functional causes of the patterns described here.

Another question we originally intended to answer was whether significant guild differences in total SDE or any of its components were to be found. The SDE of different animal guilds is expected to be affected by multiple factors. These can be behavioral; for example, certain species of frugivores are highly “wasteful” (Howe, 1980). They can be physiological, for example, if dispersal distance is a function of gut retention time, which varies across frugivores (Fuzessy et al., 2016). And they can be a combination of factors like anatomy and demography: For example, in a system in Kenya where primates constitute about 5% of the frugivorous species (Menke et al., 2012), one could naively assume that primates contribute very little to seed dispersal. But once primate fruit tree visitation rates are accounted for, primates in this system contribute to about 12% of plant–frugivore interactions. Furthermore, primates comprise ~90% of the frugivore biomass in the system, thus indicating a much more significant contribution to seed removal. Yet it is unknown whether any animal group tends to systematically provide higher or lower SDE and whether this is a product of differential quality or quantity of seed dispersal. Unfortunately, preliminary analyses revealed that the number of studies that systematically quantified SDE and its components for animals of different guilds in the same system is too small to draw any meaningful conclusion.

Future syntheses would also substantially benefit from more standardization of sampling protocols. Both components of SDE, as well as total SDE, can be estimated based on various imperfect proxies (for an overview, see our full dataset under “How was quality calculated?” and “How was quantity calculated?”). First, the proxies themselves can be improved. For example, accurate quantification of interactions, which measures the quantity subcomponent, can be much more accurate when data from multiple sources is combined (Heleno et al., 2022; Quintero et al., 2022). Quality is even more tricky to accurately estimate: the probability of establishment is the product of multiple factors that are rarely measured, such as distance from the mother tree. More standardization of methods and definitions would make studies more comparable and future syntheses more robust. Further, especially for a comparison between guilds, it is crucial to identify proxies for quality and quantity that offer minimal bias. For example, removal rates need to be recorded by means that are not likely to undercount specific guilds (e.g., animals that are less likely to trigger a camera trap), and quality should ideally be measured as establishment rates rather than, for example, gut survival rates. This need for standardization applies even more to the quality component. Quality is, in theory, the probability that a single seed survives, germinates, and grows—a standard that is very time consuming to obtain. Studies therefore rely on proxies such as gut survival, germination probability, dispersal away from the parent tree, etc. These are all expected to be correlated to some degree, though more robust downstream measures of actual establishment will be key to future syntheses of SDE (Schupp et al., 2017). Recently, van Leeuwen et al. (2022) introduced the “Extended SDE” (eSDE) concept: an extension of the SDE framework that incorporates interactions that are nominally not mutualistic (e.g., scatter hoarding, predation). In that paper, the authors also proposed a more robust and standardized approach to measuring SDE components. While applying these standards retroactively to existing literature is tricky, it offers a clear way forward that would allow more robust synthesis of SDE in the future.

Finally, our analysis focused only on studies in which the SDE of multiple frugivores dispersing a single plant species was quantified. A handful of studies did the opposite and quantified the SDE of a single animal on multiple plant species (e.g., Haurez et al., 2018). If more data are available, this approach can open the door to answering a completely different set of questions, particularly how different plant and fruit traits affect SDE and its components. We originally planned to address these questions in this study but were forced to drop it due to the very low number of studies. We are hopeful that by the time future meta‐analyses or systematic reviews are written, a sufficient number of studies taking this approach will be available.

## AUTHOR CONTRIBUTIONS

**Omer Nevo:** Conceptualization (equal); data curation (equal); formal analysis (equal); funding acquisition (equal); investigation (equal); methodology (equal); project administration (equal); writing – original draft (equal); writing – review and editing (equal). **Caitlynn Filla:** Conceptualization (equal); data curation (equal); investigation (equal); writing – review and editing (equal). **Kim Valenta:** Conceptualization (equal); investigation (equal); writing – original draft (equal); writing – review and editing (equal). **Eugene W. Schupp:** Conceptualization (equal); investigation (equal); writing – original draft (equal); writing – review and editing (equal).

## Supporting information


Figure S1

Data S1

## Data Availability

Should the manuscript be accepted, all data will be deposited in Dryad, and the data DOI will be included in the manuscript.
