# Supplementary figures and images for: What drives seed dispersal effectiveness?
Source: Ecol Evol. 2023 Aug 31;13(9):e10459. doi: 10.1002/ece3.10459 (PMC10468987; doi:10.1002/ece3.10459)

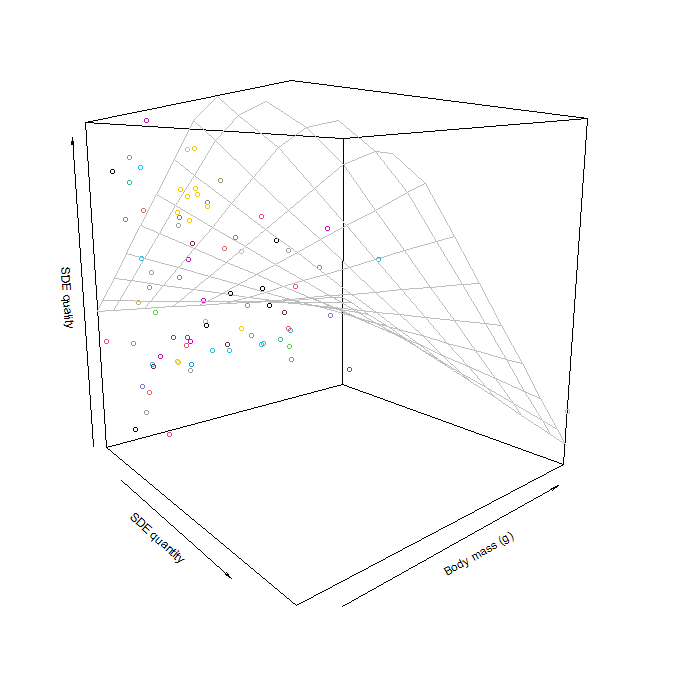

Supplement: Supplementary file 1 — Figure S1 [file ECE3-13-e10459-s002.png]
